# Characteristics of Phenolic Compounds in *Peucedanum japonicum* According to Various Stem and Seed Colors

**DOI:** 10.3390/molecules28176266

**Published:** 2023-08-27

**Authors:** Chang-Dae Lee, Hyejin Cho, Jeehyoung Shim, Gia Han Tran, Hak-Dong Lee, Kwang Hoon Ahn, Eunae Yoo, Mi Ja Chung, Sanghyun Lee

**Affiliations:** 1 Department of Plant Science and Technology, Chung-Ang University, Anseong 17546, Republic of Korea; great7321@naver.com (C.-D.L.); ghdndnlwld@naver.com (H.C.); gyoung.feb@gmail.com (J.S.); giahan1121997@gmail.com (G.H.T.); gkrehd1234@naver.com (H.-D.L.); 2 EL&I Co., Ltd., Hwaseong 18278, Republic of Korea; kevinan1122@naver.com; 3 Natural Product Institute of Science and Technology, Anseong 17546, Republic of Korea; 4 National Agrobiodiversity Center, National Institute of Agricultural Sciences, Rural Development Administration, Jeonju 54874, Republic of Korea; eung77@korea.kr; 5 Department of Food Science and Nutrition, Gwangju University, Gwangju 61743, Republic of Korea; mijachung@gwangju.ac.kr

**Keywords:** Apiaceae, caffeoylquinic acid, coastal hog fennel, color, high-performance liquid chromatography, industrial application

## Abstract

Total polyphenol and total flavonoid assays were performed to characterize the relationships between the color of *Peucedanum japonicum* (PJ) seed coat and stem and the content of phytochemical compounds. The samples were divided into two groups based on their stem and seed coat color, with each group containing 23 samples. The stem color group was subdivided into green, light red, and red, whereas the seed coat color group was divided into light brown, brown, and dark brown. In the stem color group, the light red stems exhibited the highest content of phytochemical compounds, with levels over 10% higher than those of the stems of the other colors. Moreover, among the top ten samples with the highest total polyphenol content, eight samples were light red, and the light red group also exhibited the highest total flavonoid content among the examined color groups. In terms of the seed coat color, the plants grown from dark brown seeds exhibited the highest contents of both total polyphenols and total flavonoids. In conclusion, PJ plants with dark brown seeds and light red stems contained the highest levels of phytochemical compounds. Collectively, our findings provide a valuable basis for future seed selection of PJ for pharmaceutical purposes.

## 1. Introduction

*Peucedanum japonicum* Thunb (PJ), also called ‘coastal hog fennel’, is a species of the Apiaceae family distributed along the coasts of East Asia. In Korea, this species mainly grows naturally in the central and southern coastal regions. PJ has traditionally been used to treat coughs, spasms, pain, rheumatism, asthma, and angina [1,2]. The leaves and stems of tidal-oil vegetables are called windbreak vegetables and they are eaten as wild vegetables or herbs, whereas the roots are used as medicinal ingredients [3]. Additionally, this plant has been reported to possess anti-diabetes, anti-obesity [4,5], anti-nociceptive [6], and anti-osteoporosis properties [7], in addition to reducing allergy-induced lung inflammation [8]. In traditional medicine, PJ is known to prevent stroke and vascular disease. PJ is known to contain various compounds such as rutin, 3-*O*-caffeoylquinic acid (3-CQA), 4-*O*-caffeoylquinic acid (4-CQA), 5-*O*-caffeoylquinic acid (5-CQA), and coumarins [9,10,11]. Most of the secondary metabolites of PJ possess anti-inflammatory, antioxidant, and anti-cancer properties and include compounds with multiple structures and biological activities. Therefore, several studies have sought to improve the quality of food, medicines, and cosmetics by using raw materials with a wider variety and/or higher concentrations of bioactive compounds, such as those occurring in PJ [12].

Active oxygen is involved in the progression of age-related diseases such as diabetes and cardiovascular disease. Specifically, active oxygen induces cell damage, which in turn leads to disabilities and the gradual deterioration of vascular health [13]. Mechanical evidence suggests that higher dietary antioxidant intake decreases the incidence of cardiovascular disease [14,15,16]. Moreover, increased serum antioxidant capacity improves blood sugar control [17]. Previous studies have also reported that phenolic compounds can inhibit peroxidation in the body due to their capacity to eliminate free radicals [18]. Therefore, antioxidants can prevent various diseases.

Caffeoylquinic acid (CQA), an ester of caffeine and quinic acid, is a special physiologically active metabolite derived from the phenylpropanoid biosynthesis pathway. In plants, CQA plays a role in stress tolerance [19]. These compounds have also been reported to possess antioxidant, anti-bacterial [20,21,22], and anti-cancer properties [23,24,25,26,27,28,29]. Moreover, previous studies have indicated that CQA could be applied for a wide range of therapeutic and neuroprotective purposes [30,31,32]. CQA can be acquired through the daily consumption of fruits and vegetables, and previous studies have identified a positive association between longevity and CQA intake. Other studies have confirmed that coffee consumption decreases the incidence of various degenerative diseases [33]. The presence of phenolic compounds in plants is well-known for their involvement in defense mechanisms against various threats and their contribution to plant color and pigmentation. Additionally, these compounds are responsible for creating specific odors, tastes, and colors in plants, highlighting the role of secondary metabolites in shaping the overall characteristics of plant species [34,35,36,37].

Therefore, the study investigated the correlation between the total polyphenol content (TPC) and total flavonoid content (TFC) of PJ and its stem and seed coat color. Furthermore, changes in the contents of 3-CQA, 4-CQA, and 5-CQA were also confirmed by quantifying compounds using high-performance liquid chromatography (HPLC).

## 2. Results and Discussion

Interestingly, the stem and seed colors of PJ were linked to changes in the content of highly functional compounds in this plant. The antioxidant properties of PJ have been linked to its rich content of polyphenols such as flavonoids [10]. Flavonoids have been reported to provide defense against free radicals [38]. Table 1 shows the TPC and TFC of this plant. Plants contain a wide variety of bioactive chemical compounds. Among them, polyphenols are aromatic compounds with two or more phenolic hydroxyl groups, which readily bind to proteins and other compounds [39]. Flavonoids have been reported to possess many beneficial effects, including antioxidant, anti-cancer, anti-inflammatory, and visual enhancement properties [40]. And the presence of phenolic compounds in plants is well-known for their involvement in defense mechanisms against various threats and their contribution to plant colors. Additionally, these compounds are responsible for creating specific odors, tastes, and colors in plants, highlighting the role of secondary metabolites in shaping the overall characteristics of plant species. In line with this, our study focused on one specific phenolic compound called CQA [34,35,36,37]. In Table 1, it is observed that PJ-6 displays the highest TPC among the samples, while PJ-16 exhibits the second-highest content, indicating variations in the phenolic composition of the studied PJ samples.

**Figure 1:**
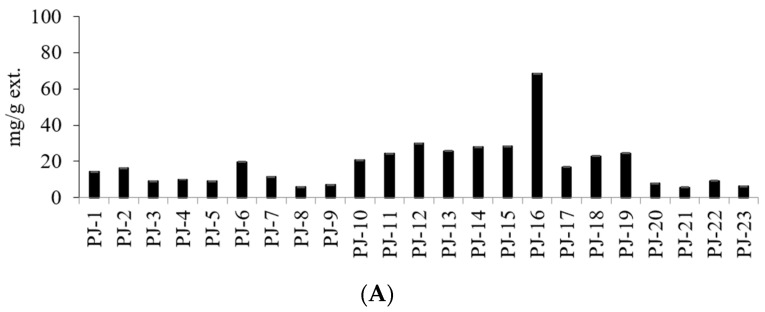

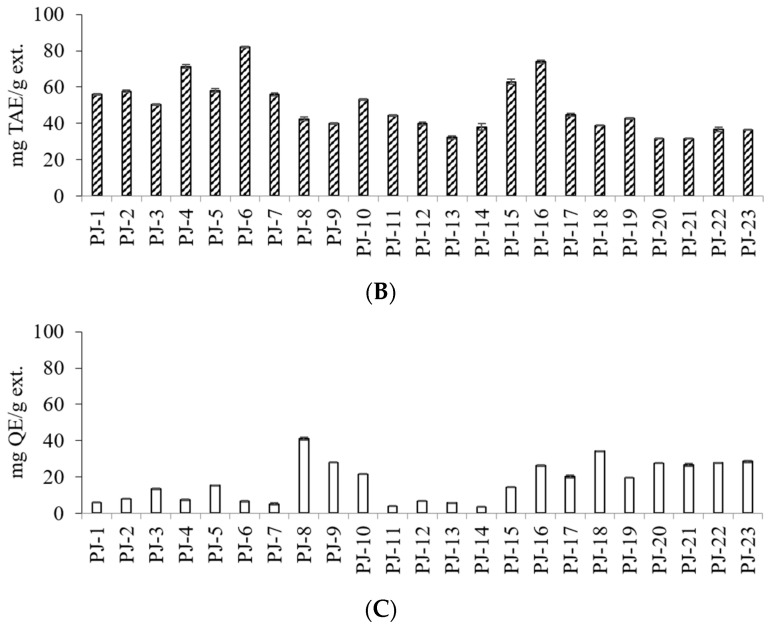
Comparison of (**A**) total CQA, (**B**) TPC, and (**C**) TFC.

In this study, we characterized the TPC of various PJ extracts. Interestingly, there was a significant difference in the content of post-flavonoid compounds depending on the plant’s stem and seed color. For TPC, the PJ-16 extract showed the highest content (81.91 mg/mL). In contrast, PJ-21 exhibited the lowest polyphenol content (31.48 mg/mL). The PJ-8 extract had the highest TFC, reaching a concentration of 40.98 mg/mL. In contrast, the PJ-7 samples exhibited the lowest TFC (5.16 mg/mL). This study also assessed the changes in the TPC and TFC according to stem and seed color (Figure 1). This was likely because the lower values obtained from the analysis were expressed in units of glycolic acid used as the standard, whereas direct measurements of phenol absorbance values were expressed in units of standard.

**Figure 2:**
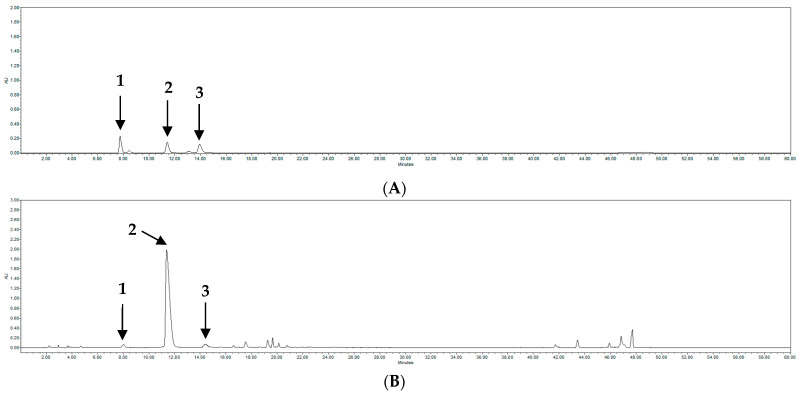
HPLC chromatograms of (**A**) CQA derivatives [(**1**) 5-CQA, (**2**) 3-CQA, and (**3**) 4-CQA] and (**B**) PJ-16.

Among other non-flavonoid natural compounds, CQA is a phenolic compound that commonly occurs in plants including coffee [41,42]. According to the existing literature, CQA possesses antioxidant [43,44], anti-mutagenic [45], and immunomodulatory properties [46], in addition to its property of decreasing the levels of adrenocortical stimulating hormone [47]. Therefore, the amounts of CQA-5 (**1**), CQA-3 (**2**), and CQA-4 (**3**) present in each sample were calculated from the calibration curve. The quantitative analysis results of PJ extracts are summarized in Table 2 and Figure 1. The contents of these compounds were found to vary depending on the seed skin and the color of the seed. The HPLC chromatogram of CQA is shown in Figure 2. PJ extract is known to contain a variety of polyphenol compounds, and the main flavonoid compounds are known as CQA-5 (**1**), CQA-3 (**2**), and CQA-4 (**3**), among others. The HPLC quantitative analysis results confirmed that there were variations in the content of CQA depending on the color of the stems (green, light red, and red) and the seeds (light brown, brown, and dark brown). PJ-16 exhibited the highest CQA-3 (**2**) content (61.73 mg/mL), in addition to high TPC and TFC. In contrast, the total CQA content of the PJ-8 samples was low. Similarly to PJ-16, the PJ-6 sample exhibited CQA levels that were consistent with those of other red stem samples. The PJ-6 sample exhibited a higher TPC than PJ-16, whereas its total CQA and TFC were not high. Most of the compound content in the samples analyzed via HPLC were similar (Table 2). However, the PJ-16 sample exhibited a difference of more than decuple compared with the sample with the lowest content. Markedly high levels of CQA-3 (**2**) were identified in the sample and the observed differences were large. However, this was likely because the contents of CQA-4 (**3**) and CQA-5 (**1**) were not significantly different. Other studies have demonstrated that CQA-3 (**2**) provides a mechanical basis for controlling microglia-dependent reactive oxygen species (ROS) production and excitotoxicity in the central nervous system via c-Src blockade, which may help in neurodegenerative diseases associated with ROS-induced nerve damage. Therefore, future studies should focus on the effects of PJ extracts at the gene expression level to explore the molecular mechanisms underlying their therapeutic properties.

In conclusion, the stem and seed color variations in PJ have a significant impact on its phytochemical composition, particularly the TPC and TFC, as well as the concentrations of specific compounds such as 5-CQA, 3-CQA, and 4-CQA. These observations highlight the role of stem and seed color as determining factors for the chemical profile of PJ. These findings have important implications from a pharmaceutical perspective, as they provide insights into the potential health benefits and diverse applications of PJ based on its varying phytochemical composition.

Indeed, our investigation extends beyond the scope of color-related metabolite variations. We delve into a broader spectrum of plant characteristics, encompassing morphological traits such as stem and seed color, which could potentially exert an influence on these content changes. By establishing potential correlations between these distinctive features and the fluctuation in metabolite content, our intent is to illuminate any potential interconnectedness. This holistic approach, as elucidated in our conclusion, underscores the comprehensive nature of our study, providing a nuanced understanding of the intricate factors that mold metabolite variation. Our findings propose an intriguing association, suggesting that the variations in stem and seed color interact with a diverse range of secondary metabolite production. This interplay contributes to the intricate tapestry of metabolite diversity within PJ, adding further depth to our comprehension of this remarkable plant’s characteristics.

## 3. Material and Methods

### 3.1. Plant Materials

**Figure 3:**
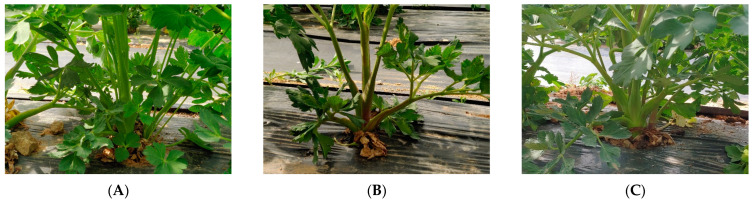
Photographs of PJ plants (**A**) PJ-20 with green stems, (**B**) PJ-16 with red stems, and (**C**) PJ-11 with light red stems.

**Figure 4:**
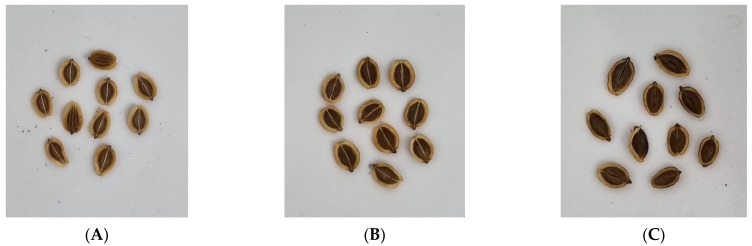
Photographs of PJ seeds (**A**) PJ-15 with light brown seeds, (**B**) PJ-9 with brown seeds, and (**C**) PJ-5 with dark brown seeds.

The seeds of 23 PJ (*Peucedanum japonicum* Thunb) specimens were obtained from 21 accessions from the National Agrobiodiversity Center of Rural Development Administration and two accessions from EL&I Co., Ltd., Hwaseong, Korea. The collected seeds were immersed in running water for 24 h for the dormancy release to remove dormant substances, and then treated at a low temperature of 3 °C for a week in a wet towel. The treated seeds were sown in March 2019. Leaf samples were collected and dried from young shoots grown to a length of 15–20 cm during 12–16 May 2020. The seeds were harvested from 12 August 2020, to 10 September 2020, and from 18 August 2021, to 1 October 2021, by biennial and triennial, respectively (Figure 3 and Figure 4). PJ specimens were identified by EL&I supported by the “Cooperative Research Program for Agriculture Science and Department”. Voucher specimens were deposited at the herbarium of the National Agrobiodiversity Center of Rural Development Administration of Korea.

### 3.2. Plant Identification

The phenotype characteristic investigation was divided into two parts: measurement and observation. Stem and seed colors were investigated by observation, and sensory evaluation was conducted by humans. In the case of stem color, cross-checking was performed by two or more people, and in the case of seed color, after arranging according to gradient of seed color, representative colors were selected and evaluated by dividing them into three groups.

### 3.3. Phenological Status

In the case of major crops, there is a uniformity in cultivation, so it is possible to study the growth stage by date. On the other hand, in the case of minor crops such as medicinal crops, cultivation is not uniform, and it is relatively difficult to investigate and study by specifying the date. In particular, in the case of PJ, it is a plant that blooms in 2 or 3 years, and the flowering period may show a difference of 1 year. Differences in phenotypes by growth stage are also related to the biosynthetic process of metabolites. In the case of PJ, differences in stem color by growth stage could be observed, and in this paper, research was conducted based on stem color data during the flowering period.

### 3.4. Seed Collection and Processing

PJ is an outcrossing plant, a biennial or triennial, producing seeds once every 2 or 3 years. In order to prevent natural crossing with other resources, isolation nets were placed during flowering and seeds were harvested individually during the ripening period. After growing a maximum of 12 individuals per accession, we secured seeds for each accession by mixing the same amount of harvested seeds for each individual.

### 3.5. Instruments and Reagents

**Figure 5:**
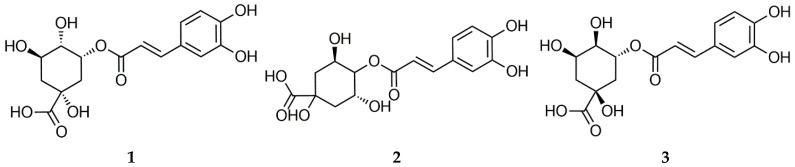
Chemical structures of (**1**) 5-CQA, (**2**) 3-CQA, and (**3**) 4-CQA.

All PJ samples were analyzed using an HPLC instrument (Waters Alliance 2695 Separation Module, Milford, MA, USA) with a photodiode array detector (Waters 996 PDA Detector, Milford, MA, USA). The setup also included pumps and autosamplers coupled with a YMC Pack Pro C18 column (4.6 × 250 mm, 5 μm). HPLC-grade solvents [water, acetonitrile (ACN), and methanol (MeOH)] were purchased from J. T. Baker (Phillipsburg, Pennsylvania). Ethanol (EtOH) and acetic acid were purchased from Samcheon Chemical (Pyeongtaek, Korea). Gallic acid, quercetin, 5-CQA (**1**), 3-CQA (**2**), and 4-CQA (**3**) were obtained from the Natural Product Institute of Science and Technology (www.nist.re.kr (accessed on 1 March 2022)), Anseong, Republic of Korea (Figure 5).

### 3.6. Sample Extraction and Preparation

To compare the CQA, TPC, and TFC, aerial parts were extracted, and the samples were divided into groups based on stem and seed part color. PJ was dried using a reflux cooling extraction method and PJ was sequentially extracted three times for 3 h using EtOH. The sample was dried using a vacuum concentrator to obtain 1.0 to 2.0 g of the extract followed by analyses using HPLC-PDA system. For quantitative evaluation of the three contents (CQA, TPC, and TFC) in the PJ extract, the extract was dissolved in MeOH, sonicated for 30 min, and filtered through a 0.45 μm polyvinylidene fluoride (PVDF) membrane filter. A stock solution was also prepared and the CQA, TPC, and TFC of all samples were measured using a test curve.

### 3.7. Analysis of TPC

The polyphenol contents of PJ were measured as described in a previous study [48,49]. Briefly, 60 μL the extract was mixed with 40 μL of 2N Folin–Ciocalteu phenol reagent (St. Louis, Sigma-Aldrich, Burlington, MA, USA). Afterward, 100 μL of 7.5% sodium carbonate solution was added to the mixture and incubated in the dark for 30 min. The absorbance of the samples was measured using a microplate reader (Epoch, BioTek, Winooski, VT, USA) at a 760 nm wavelength. Finally, a calibration curve was prepared using gallic acid as a reference and the TPC was quantified.

### 3.8. Analysis of TFC

The PJ extract was analyzed using the method described in a previous study [48] with some modifications. Briefly, 100 μL of 1 mg/mL extract was mixed with 100 μL of 2% AlCl_3_. The solution was then incubated for 10 min and the absorbance was measured at 430 nm using a microplate reader (Epoch, BioTek, Winooski, VT, USA). A calibration curve was created using quercetin as the standard compound and the TFC was determined.

### 3.9. HPLC Conditions

The quantitative analysis of compounds in PJ was performed using a reverse-phase HPLC-PDA system. Chromatographic separation was performed using a YMC Pack Pro C18 column (4.6 × 250 mm, 5 μm). The analyses were conducted using a gradient of 0.5% acetic acid in water (A) and ACN (B). The elution system was as follows: 0–10 min 90% solution A; 40 min 30% solution A; 45 min 0% solution A; 50 min 90% solution A; 60 min solution 90% A. The temperature of the column was maintained at 40 °C. The injection volume was 10 μL, the flow rate was set to 1.0 mL/min, and the wavelength of the PDA detector was set to 310 nm.

### 3.10. HPLC Calibration Curve

The compounds 5-CQA (**1**), 3-CQA (**2**), and 4-CQA (**3**) were dissolved in MeOH (1 mg/mL) as a standard solvent. The working solution used to prepare the calibration curve was prepared by continuously diluting the standard solution to the desired concentration. PJ samples were dissolved in MeOH (30 mg/mL). The standard solution and the sample solution were filtered using a 0.45 to μm PVDF filter. The CQA and TPC of the PJ extracts were then determined from the calibration curve by HPLC (Table 3). The calibration function of the three compounds was calculated as the peak area (Y), concentration (X, μg/10 μL), and average value (n = 5) ± standard deviation (SD).

### 3.11. Statistical Analysis

Results were subjected to one-way analysis of variance (ANOVA) using SAS analytical software. The values were expressed as the mean ± SD.

## Figures and Tables

**Table 1:** Comparison of TPC and TFC by stem and seed color of PJ.

Sample No.	Stem Color ^z^	Seed Color ^y^	TPC (mg TAE/g ext.)		TFC (mg QE/g ext.)	
PJ-1	G	DB	56.01 ± 0.37	F	5.93 ± 0.22	P
PJ-2	G	LB	57.60 ± 0.55	E	7.97 ± 0.11	M
PJ-3	G	B	50.20 ± 0.44	H	13.35 ± 0.25	L
PJ-4	LR	B	71.30 ± 1.11	C	7.31 ± 0.28	N
PJ-5	LR	DB	58.00 ± 0.94	E	15.37 ± 0.14	J
PJ-6	R	B	81.91 ± 0.32	A	6.59 ± 0.31	O
PJ-7	LR	B	55.99 ± 0.69	F	5.16 ± 0.42	Q
PJ-8	LR	LB	42.48 ± 1.19	J	40.98 ± 0.89	A
PJ-9	LR	B	39.99 ± 0.47	K	28.04 ± 0.04	DE
PJ-10	LR	LB	53.15 ± 0.47	G	21.61 ± 0.28	G
PJ-11	LR	DB	44.38 ± 0.38	I	3.80 ± 0.16	R
PJ-12	LR	B	40.04 ± 0.82	K	6.71 ± 0.14	O
PJ-13	LR	LB	32.21 ± 0.87	O	5.78 ± 0.04	O
PJ-14	LR	LB	38.00 ± 1.79	L	3.51 ± 0.19	P
PJ-15	LR	LB	62.94 ± 1.52	D	14.25 ± 0.22	K
PJ-16	R	DB	73.88 ± 0.81	B	26.34 ± 0.42	F
PJ-17	LR	B	44.70 ± 0.77	I	20.29 ± 0.73	H
PJ-18	LR	LB	38.86 ± 0.05	N	34.16 ± 0.14	B
PJ-19	LR	B	42.77 ± 0.43	J	19.61 ± 0.26	I
PJ-20	G	B	31.53 ± 0.13	O	27.58 ± 0.19	E
PJ-21	G	B	31.48 ± 0.18	O	30.91 ± 0.37	C
PJ-22	LR	DB	36.67 ± 1.12	M	27.73 ± 0.22	E
PJ-23	LR	DB	36.30 ± 0.20	M	28.51 ± 0.59	D
One-way ANOVA ^x^				Significance		
		Assay		F	*p*	
		TPC		1035.22	<0.0001	
		TFC		3088.67	<0.0001	

^z^ R = red, G = green, LR = light red, ^y^ B = brown, LB = light brown, DB = dark brown. ^x^ Mean separation within by Duncan’s multiple range test at *p* < 0.05.

**Table 2:** Content of (**1**) 5-CQA, (**2**) 3-CQA, and (**3**) 4-CQA.

Sample No.	Stem Color ^z^	Seed Color ^y^	Contents (mg/g ext.)						
			1		2		3		Total
PJ-1	G	DB	0.53 ± 0.01	U	13.39 ± 0.01	I	0.21 ± 0.01	V	14.13 ± 0.03
PJ-2	G	LB	1.24 ± 0.01	P	12.45 ± 0.16	J	2.63 ± 0.01	L	16.32 ± 0.18
PJ-3	G	B	0.78 ± 0.01	T	6.92 ± 0.00	M	1.26 ± 0.01	Q	8.96 ± 0.02
PJ-4	LR	B	1.49 ± 0.00	N	6.65 ± 0.00	N	1.71 ± 0.00	O	9.85 ± 0.00
PJ-5	LR	DB	1.29 ± 0.01	O	5.64 ± 0.01	P	2.02 ± 0.01	N	8.95 ± 0.03
PJ-6	R	B	4.40 ± 0.03	D	10.44 ± 0.02	L	4.81 ± 0.04	D	19.65 ± 0.09
PJ-7	LR	B	2.50 ± 0.01	K	6.47 ± 0.01	O	2.35 ± 0.00	M	11.32 ± 0.02
PJ-8	LR	LB	1.13 ± 0.00	Q	4.09 ± 0.01	S	0.61 ± 0.01	T	5.83 ± 0.02
PJ-9	LR	B	1.04 ± 0.00	R	5.36 ± 0.01	Q	0.81 ± 0.01	S	7.21 ± 0.02
PJ-10	LR	LB	3.40 ± 0.02	H	13.85 ± 0.05	H	3.39 ± 0.01	I	20.64 ± 0.08
PJ-11	LR	DB	3.20 ± 0.01	I	18.17 ± 0.04	D	3.10 ± 0.01	J	24.47 ± 0.06
PJ-12	LR	B	5.29 ± 0.02	B	19.65 ± 0.05	B	5.01 ± 0.01	C	29.95 ± 0.08
PJ-13	LR	LB	3.53 ± 0.02	F	17.71 ± 0.03	E	4.47 ± 0.05	G	25.71 ± 0.1
PJ-14	LR	LB	4.25 ± 0.02	E	19.17 ± 0.08	C	4.59 ± 0.01	F	28.01 ± 0.11
PJ-15	LR	LB	6.18 ± 0.01	A	16.73 ± 0.06	F	5.29 ± 0.01	B	28.2 ± 0.08
PJ-16	R	DB	2.40 ± 0.01	L	61.73 ± 0.05	A	4.24 ± 0.01	H	68.37 ± 0.07
PJ-17	LR	B	2.76 ± 0.01	J	11.15 ± 0.05	K	2.97 ± 0.01	K	16.88 ± 0.07
PJ-18	LR	LB	3.44 ± 0.02	G	14.70 ± 0.06	G	4.70 ± 0.02	E	22.84 ± 0.1
PJ-19	LR	B	5.16 ± 0.01	C	13.83 ± 0.04	H	5.47 ± 0.03	A	24.46 ± 0.08
PJ-20	G	B	1.49 ± 0.01	N	5.61 ± 0.01	P	0.82 ± 0.03	P	7.92 ± 0.05
PJ-21	G	B	1.05 ± 0.01	R	4.05 ± 0.02	S	0.56 ± 0.01	U	5.66 ± 0.04
PJ-22	LR	DB	2.01 ± 0.03	M	5.59 ± 0.02	P	1.68 ± 0.02	P	9.28 ± 0.07
PJ-23	LR	DB	1.00 ± 0.01	S	4.28 ± 0.00	R	0.87 ± 0.02	R	6.15 ± 0.03
One-way ANOVA ^x^				Significance					
		Compound		F			*p*		
		5-CQA		41,949.0			<0.0001		
		3-CQA		183,315			<0.0001		
		4-CQA		27,746.1			<0.0001		

^z^ R = red, G = green, LR = light red, ^y^ B = brown, LB = light brown, DB = dark brown. ^x^ Mean separation within by Duncan’s multiple range test at *p* < 0.05.

**Table 3:** Calibration curve of (**1**) 5-CQA, (**2**) 3-CQA, and (**3**) 4-CQA.

Compound	t_R_	Calibration Equation ^z^	Correlation Factor, *r*^2 y^
**1**	7.9	Y = 9894.8 X − 63,398	0.9992
**2**	11.6	Y = 23,864 X − 1,000,000	0.9997
**3**	14.4	Y = 7430.8 X − 120,529	0.9992

^z^ Y = peak area, X = concentration of standards (µg/mL), ^y^ *r*^2^ = correlation coefficient based on three data points in the calibration curves.

## Data Availability

Not applicable.
